# Associations of body morphology and body composition with swimming performance in male and female youth swimmers: an exploratory cross-sectional study

**DOI:** 10.3389/fphys.2026.1963914

**Published:** 2026-09-10

**Authors:** Zhanyang He, Sujing Su, Xujun Liu, Xun Jin, Cuimei Shen, Houwei Zhu, Gongju Liu

**Affiliations:** 1 School of Sport Science, Beijing Sport University, Beijing, China; 2 College of Physical Education and Health Sciences, Zhejiang Normal University, Jinhua, China; 3 Key Laboratory of Aquatic Sports Science, General Administration of Sport, Zhejiang College of Sports, Hangzhou, China; 4 Department of Business Administration, Shanghai University of Finance and Economics Zhejiang College, Jinhua, China

**Keywords:** body composition, body morphology, hand area, swimming performance, youth swimmers

## Abstract

**Objective:**

To investigate the associations of body morphology and body composition with swimming performance in male and female provincial youth swimmers.

**Methods:**

This cross-sectional study included 49 swimmers: 25 males (FINA points: 582 ± 66; age: 12.5 ± 0.7 years; height: 162.3 ± 6.6 cm; weight: 47.4 ± 6.9 kg) and 24 females (FINA points: 620 ± 84; age: 11.8 ± 0.8 years; height: 156.4 ± 6.1 cm; weight: 43.5 ± 5.1 kg). Competitive performance was represented by each swimmer’s highest FINA point score. BMI, body proportions, body fat percentage, and hand and foot areas were assessed. Sex-stratified correlation and exploratory regression analyses were performed with false discovery rate correction and formal interaction testing.

**Results:**

Among female swimmers, FINA points were positively correlated with age, BMI, hand area, and foot area after FDR correction, whereas no corrected associations were observed among males. In the primary adjusted female model, hand area remained positively associated with FINA points; however, this estimate was attenuated after influential observations were excluded (β = 0.370, p = 0.052). Neither the sex × BMI nor the sex × hand area interaction was significant.

**Conclusion:**

In this exploratory sample, hand area showed a positive adjusted association with FINA points among female swimmers, but the estimate was sensitive to influential observations. These findings are hypothesis-generating and require confirmation in larger independent samples.

## 1 Introduction

Adolescence is a critical period in which the development of swimming-specific abilities, improvements in competitive performance, and long-term athletic development occur concurrently. Athletes continue to undergo substantial growth and biological maturation during this period, and similar chronological ages do not necessarily indicate comparable levels of physical development, neuromuscular capacity, or training adaptation. The International Olympic Committee consensus statement on youth athletic development therefore, emphasises that athletic potential should be evaluated within an individualised and dynamic developmental framework (Bergeron et al., 2015). Systematic reviews of youth swimmers further indicate that competitive performance cannot be explained by a single physical characteristic; rather, it is jointly associated with strength and power, lean body mass, aerobic and anaerobic capacity, and technical and biomechanical factors (Abbott et al., 2021; Price et al., 2024). From a biophysical perspective, the ability to generate effective propulsion while controlling hydrodynamic drag is central to translating these factors into race performance (Barbosa et al., 2010). Analyses of the physical basis of youth swimming performance should therefore account for growth and maturation, training experience, and swimming-specific technical context.

Body morphology and body composition provide an important structural basis for swimming propulsion, drag control, and stroke kinematics. A systematic review of anthropometric research in youth swimmers showed that greater height and arm span, together with higher body mass and lean body mass, were relatively consistently associated with longer stroke length, a higher stroke index, and better freestyle performance, although the evidence was less consistent for backstroke, breaststroke, and butterfly (Alves et al., 2022). In male adolescent swimmers, arm span has been identified as an important anthropometric correlate of 100 m freestyle performance, although technical variables such as stroke rate and stroke index explained more variance (Lätt et al., 2010). Height, arm span, lean body mass, and upper-arm muscle area have also been associated with upper-limb propulsive force (Moura et al., 2014). Transverse body dimensions, including shoulder and pelvic breadth, may also be relevant to swimming hydrodynamics because they contribute to frontal body morphology and have been associated with passive drag in young swimmers (Cortesi et al., 2020). Composite indices derived from these dimensions may therefore provide descriptive information on relative body breadth, although they should not be regarded as direct measures of hydrodynamic resistance. These findings suggest that conventional anthropometric measures characterise overall body size and muscularity, but their associations with performance may vary according to stroke, distance, maturational stage, and technical proficiency.

Compared with general measures of body size, hand and foot areas may more directly reflect the structural requirements of the propulsive interface in swimming. Computational fluid dynamics studies have shown that hydrodynamic forces acting on the hand depend closely on hand orientation, direction of motion, angle of attack, and relative water velocity (Sato and Hino, 2013). In trained adult swimmers, increasing hand-paddle surface area alters stroke rate, stroke length, and the energetic cost of swimming at a fixed speed, although the benefit does not increase indefinitely with surface area (Crocker et al., 2021). Research on lower-limb propulsion likewise indicates that the feet and lower legs contribute to vortex formation and changes in water momentum, while fins of different areas and stiffnesses alter propulsive efficiency and distance travelled per kick (Andersen and Sanders, 2018; Zamparo et al., 2006). In youth swimmers, hand area may change with seasonal training and growth in parallel with in-water force, stroke kinematics, and swimming speed (Santos et al., 2024). Hand and foot areas, together with other anthropometric characteristics, also show age-, performance-level-, or sex-related differences (Morais et al., 2013). However, existing evidence has largely focused on a single stroke, short-distance tests, or surrogate measures of propulsion. Whether hand and foot areas explain standardised race performance after accounting for age, training experience, and general body size has not been directly examined in high-level youth swimmers.

Sex and biological maturation may further shape the context in which physical characteristics are associated with swimming performance. Large competition datasets indicate that the performance gap between male and female youth swimmers begins to widen at approximately 10 years of age, coinciding with the pubertal period (Senefeld et al., 2019). Longitudinal research has also shown that developmental trajectories in anthropometry, maximal swimming velocity, and stroke index during puberty are related to subsequent competitive level (Post et al., 2024). Characteristics such as height, arm span, and lean body mass may mediate associations between biological maturation and propulsive force or sprint performance (Matos et al., 2025). In female youth swimmers, menarcheal status is also related to the developmental trajectories of hand length, body size, strength, and performance across different race distances (López-Plaza et al., 2025). Sex-stratified analyses may therefore help describe patterns of association within different developmental contexts, but the presence of true sex-related moderation requires confirmation through formal interaction testing.

Accordingly, this study aimed to investigate the associations of body morphology and body composition with standardised competitive performance (FINA points) in male and female provincial youth swimmers, with particular emphasis on swimming-specific anthropometric measures such as hand and foot areas. We proposed the following hypotheses: (1) body morphology and body composition would be significantly associated with competitive performance in youth swimmers; (2) after adjustment for age and training duration, selected physical characteristics would independently explain differences in competitive performance; and (3) because male and female swimmers differ in growth, maturation, and body composition, the patterns of association between physical characteristics and competitive performance might be sex-specific.

## 2 Methods

### 2.1 Participants

This exploratory cross-sectional study was designed and reported in accordance with the STROBE statement (von Elm et al., 2007). Participants were actively training members of a provincial youth swimming team. The inclusion criteria were as follows: (1) registration with and continuous training in the provincial youth swimming team; (2) complete and verifiable information on sex, age, and training duration; (3) at least 1 valid official race result or corresponding FINA point score; and (4) completion of the principal body morphology and body composition assessments. Duplicate records, unverifiable race results, FINA point scores of 0, and records with missing key study variables were excluded. Physiologically plausible extreme values that passed data verification were not automatically removed; instead, their influence was evaluated through regression diagnostics and sensitivity analyses. The final sample comprised 49 swimmers, including 25 males and 24 females. The study protocol was reviewed and approved by the Ethics Committee of Zhejiang Normal University (approval no. ZSRT2025108). Written informed consent was obtained from all athletes and their legal guardians before study commencement.

### 2.2 Data collection and processing

#### 2.2.1 Competitive performance data

World Aquatics Swimming Points (formerly FINA Points and hereafter referred to as FINA points) were used as the standardised measure of competitive performance. According to the 2026 World Aquatics Swimming Points Table, points were calculated as P = 1000 × (B/T)³, where T is the race time (s) and B is the 1000-point benchmark time for the corresponding sex, stroke, distance, and pool length. The resulting point score was truncated to an integer, with a higher score indicating better performance relative to the corresponding event benchmark (World Aquatics, 2026). Event-specific inferential analyses were not undertaken because further subdivision by sex, stroke, and distance would have resulted in very small subgroups and potentially unstable estimates.

Competition results were obtained from the official results database for national-level youth competitions. The included events were the 50 m freestyle, breaststroke, butterfly, and backstroke; the 100 m freestyle, breaststroke, butterfly, and backstroke; the 200 m freestyle, breaststroke, and individual medley; and the 400 m, 800 m, and 1500 m freestyle. Each result was converted using the corresponding 2026 points table. For each swimmer, the highest FINA point score among all recorded events was selected as the primary outcome to represent the individual’s best standardised competitive performance. This measure facilitates comparison across events but is not equivalent to performance in a specific stroke or distance.

#### 2.2.2 Body morphology and body composition data

Body morphology and body composition data were obtained during field-based assessments of provincial youth swimmers. All assessments were conducted in Zhejiang, China, between June and September 2025 within the national-team testing programme and in accordance with the national-team testing protocol under standardized conditions.

The analysed variables were age, training duration, height, body mass, body mass index (BMI), streamline index (%), trunk-to-leg-length ratio, arm-span-to-height ratio, body fat percentage, hand area, and foot area. Age was calculated as decimal age from the date of birth and assessment date, and training duration was obtained from training records. Operational definitions of BMI and the body-proportion measures are presented in **Table 1**, and the image-based measurement procedures for hand and foot areas are described in Section 2.2.3.

**Table 1:** Operational definitions of body morphology and body composition measures.

Measure	Operational definition or method of acquisition	Unit
BMI	Body mass (kg)/height² (m²)	kg/m²
Streamline index (%)	The streamline index was derived from the national-team testing protocol and calculated as [(shoulder breadth + bi-iliac breadth)/height] × 2. Higher values indicate greater combined shoulder and bi-iliac breadth relative to stature. The index was treated as an exploratory composite measure of body morphology rather than a direct measure of hydrodynamic drag.	%
Trunk-to-leg-length ratio	Trunk length/leg length (specific anatomical landmarks are defined in the testing protocol)	Dimensionless
Arm-span-to-height ratio	Arm span (cm)/height (cm)	Dimensionless

Body fat percentage was assessed using an InBody 270 multifrequency segmental bioelectrical impedance analyser (InBody Co., Ltd., Seoul, Republic of Korea). This device has shown high relative agreement and test-retest reliability in children aged 10–12 years, although absolute estimates of fat mass and lean body mass may be subject to sex-related systematic bias (Larsen et al., 2021). Body fat percentage was therefore treated as a continuous comparative measure obtained under a standardised protocol rather than as an individual clinical diagnostic measure.

#### 2.2.3 Measurement of hand and foot areas

**Figure 1:**
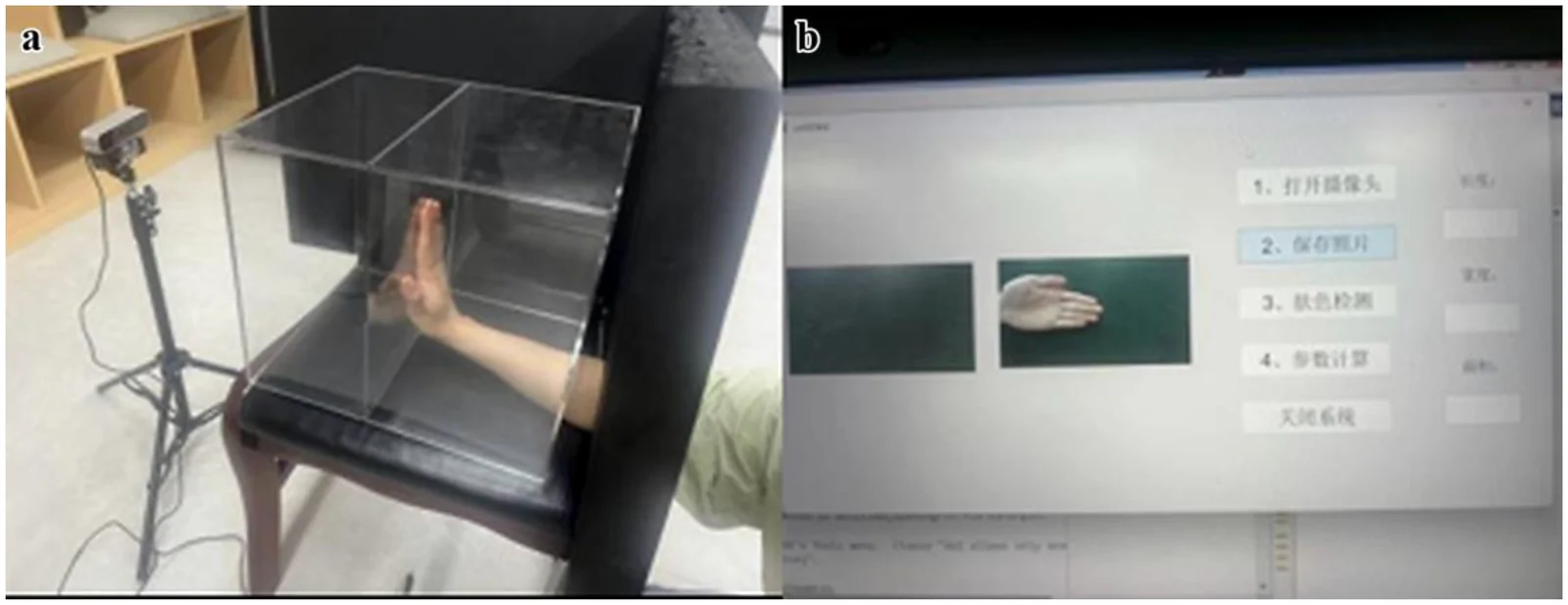
Two-dimensional image-based system for measuring hand and foot areas. **(a)** Experimental apparatus comprising a monocular industrial camera, transparent acrylic measurement plate, and black background panel; **(b)** software interface for image acquisition and calculation of hand and foot areas.

Hand and foot areas were measured using a two-dimensional image-acquisition system based on a monocular industrial camera. The system comprised a monocular industrial camera, a transparent acrylic measurement plate, a standard-sized calibration object, and a black background panel. Each swimmer placed the palm and plantar surface naturally against the transparent plate, with the fingers or toes naturally extended. The optical axis of the camera was perpendicular to the measurement plane, and the camera-to-plane distance was held constant to minimise perspective and scale variation. Two-dimensional photogrammetry has been used to assess anthropometric measures such as hand area in swimmers, and image-based measurements show high agreement with direct hand anthropometry (Morais et al., 2013, 2019; Patel et al., 2018) (**Figure 1**).

Images were processed offline using MATLAB (MathWorks Inc., Natick, MA, USA). Raw images underwent grayscale conversion, contrast enhancement, threshold segmentation, and binarisation, after which the closed contour of the palm or plantar surface was extracted. Visible holes and non-target connected components were processed using consistent rules, and the number of pixels within the target region was then calculated.

The actual projected area was calculated as A = Ntarget × k, where A is the projected palm or plantar area (cm²), Ntarget is the number of pixels in the target region, and k is the area-conversion coefficient (cm²/pixel) derived from a standard calibration object positioned in the same measurement plane. The calibration object and target were placed in the same plane to minimise scale errors arising from differences in distance. The left and right palms and plantar surfaces were measured separately, and the bilateral mean was used as the final hand-area and foot-area measure. These measures represent the potential projected area of the distal limbs interacting with the water and are not direct measures of in-water propulsive force.

### 2.3 Statistical analysis

Continuous variables are presented as mean ± standard deviation (SD). Differences between male and female swimmers were examined using Welch’s independent-samples t-test to reduce the effect of unequal variances on statistical inference (Welch, 1947). Hedges’ g, corrected for small-sample bias, and its 95% confidence interval (CI) were also reported (Hedges and Olkin, 1985).

To test Hypothesis 1, Pearson correlation coefficients were calculated separately for male and female swimmers between FINA points and age, training duration, BMI, streamline index, trunk-to-leg-length ratio, arm-span-to-height ratio, body fat percentage, hand area, and foot area. Spearman rank correlations were used as distribution-robust sensitivity analyses. The 95% CIs for Pearson’s r were calculated using Fisher’s z transformation. Two-sided p values were based on 5000 random permutations and calculated as (b + 1)/(m + 1), where b is the number of permutations with an absolute test statistic at least as large as the observed value and m is the total number of permutations (Phipson and Smyth, 2010). The Benjamini-Hochberg procedure was applied separately to Pearson and Spearman correlations across the same 9 measures within each sex to control the false discovery rate (FDR), and q values were reported (Benjamini and Hochberg, 1995).

To test Hypothesis 2, ordinary least-squares linear regression was used to estimate the adjusted associations of each physical measure with FINA points. Age and training duration were included as basic covariates, and each candidate physical measure was initially entered in a separate model. Regression parameters were estimated using HC3 heteroskedasticity-robust covariance estimates to improve finite-sample inference in the presence of a small sample and potential heteroskedasticity (Mackinnon and White, 1985). Unstandardised coefficients (B), standardised coefficients (β), 95% CIs, and two-sided p values were reported. Benjamini-Hochberg FDR correction was applied to tests of the candidate measures within each sex.

To explore the incremental explanatory value of key physical measures beyond age and training experience, exploratory sequential models were constructed. Model M1 included age and training duration, M2 additionally included BMI, and M3 additionally included hand area. Hand area was added after the preliminary correlation analysis because of its observed association with FINA points and its biomechanical relevance. Accordingly, these models were considered hypothesis-generating rather than prespecified confirmatory models. Adjacent models were compared using nested-model F-tests, and changes in R² (ΔR²) were reported. The models were not intended to establish talent-identification thresholds or individual prediction tools.

To test Hypothesis 3, models were fitted sequentially in the pooled sample: P1 included sex, age, and training duration; P2 included P1 plus BMI and hand area; and P3 included P2 plus the sex × BMI and sex × hand area interactions. Continuous variables were z-standardised before the interaction terms were constructed. In addition, after adjustment for sex, age, and training duration, the interaction between sex and each of the 9 candidate measures was tested separately, with Benjamini-Hochberg FDR correction applied across the 9 interaction tests. A sex-related moderating effect was considered statistically supported only when the corrected q value was < 0.05.

Model fit was evaluated using R², adjusted R², and root mean square error (RMSE), and internal-validation RMSE was calculated using leave-one-out cross-validation (LOOCV) (Stone, 1974). Multicollinearity was assessed using the variance inflation factor (VIF) (O'Brien, 2007). Influential observations were identified using Cook’s distance, with 4/n applied as a screening threshold. Core models were refitted after excluding flagged observations as a sensitivity analysis, whereas the primary analyses retained all valid observations that passed data verification (Cook, 1977).

All tests were two-sided. An uncorrected p value < 0.05 was considered nominally significant, and an FDR-corrected q value < 0.05 was considered significant after adjustment for multiple comparisons. Data processing and statistical analyses were performed in Python 3.11.0, primarily using NumPy 1.26.4, pandas 2.2.3, SciPy 1.14.1, and Matplotlib 3.8.2.

## 3 Results

### 3.1 Participant characteristics

A total of 49 provincial youth swimmers were included (25 males and 24 females). Male swimmers were older, taller, and heavier, had a lower body fat percentage, and had larger hand and foot areas than female swimmers (**Table 2**).

**Table 2:** Participant characteristics and descriptive statistics stratified by sex.

Variable	Male (n=25)	Female (n=24)	*p*	Hedges’ g
FINA points	582 ± 66	620 ± 84	0.082	-0.50
Age (years)	12.5 ± 0.7	11.8 ± 0.8	0.003	0.89
Training duration(years)	6.000 ± 1.958	5.458 ± 1.615	0.295	0.30
Height (cm)	162.3 ± 6.6	156.4 ± 6.1	0.002	0.91
Body mass (kg)	47.4 ± 6.9	43.5 ± 5.1	0.028	0.64
BMI (kg/m^2^)	18.0 ± 1.5	17.7 ± 1.3	0.459	0.21
Streamline index (%)	83.4 ± 4.8	82.9 ± 5.8	0.737	0.10
Trunk-to-leg-length ratio	0.893 ± 0.071	0.881 ± 0.048	0.506	0.19
Arm-span-to-height ratio	1.039 ± 0.026	1.035 ± 0.031	0.645	0.13
Body fat percentage (%)	13.3 ± 2.9	16.2 ± 2.7	< 0.001	-1.01
Hand area (cm^2^)	116.5 ± 9.3	102.5 ± 14.0	< 0.001	1.17
Foot area (cm^2^)	141.4 ± 17.1	114.7 ± 17.2	< 0.001	1.53

Data are presented as mean ± standard deviation. p-values were obtained from Welch’s independent-samples t-tests, and Hedges’ g was calculated as male minus female.

### 3.2 Correlation analysis

**Figure 2:**
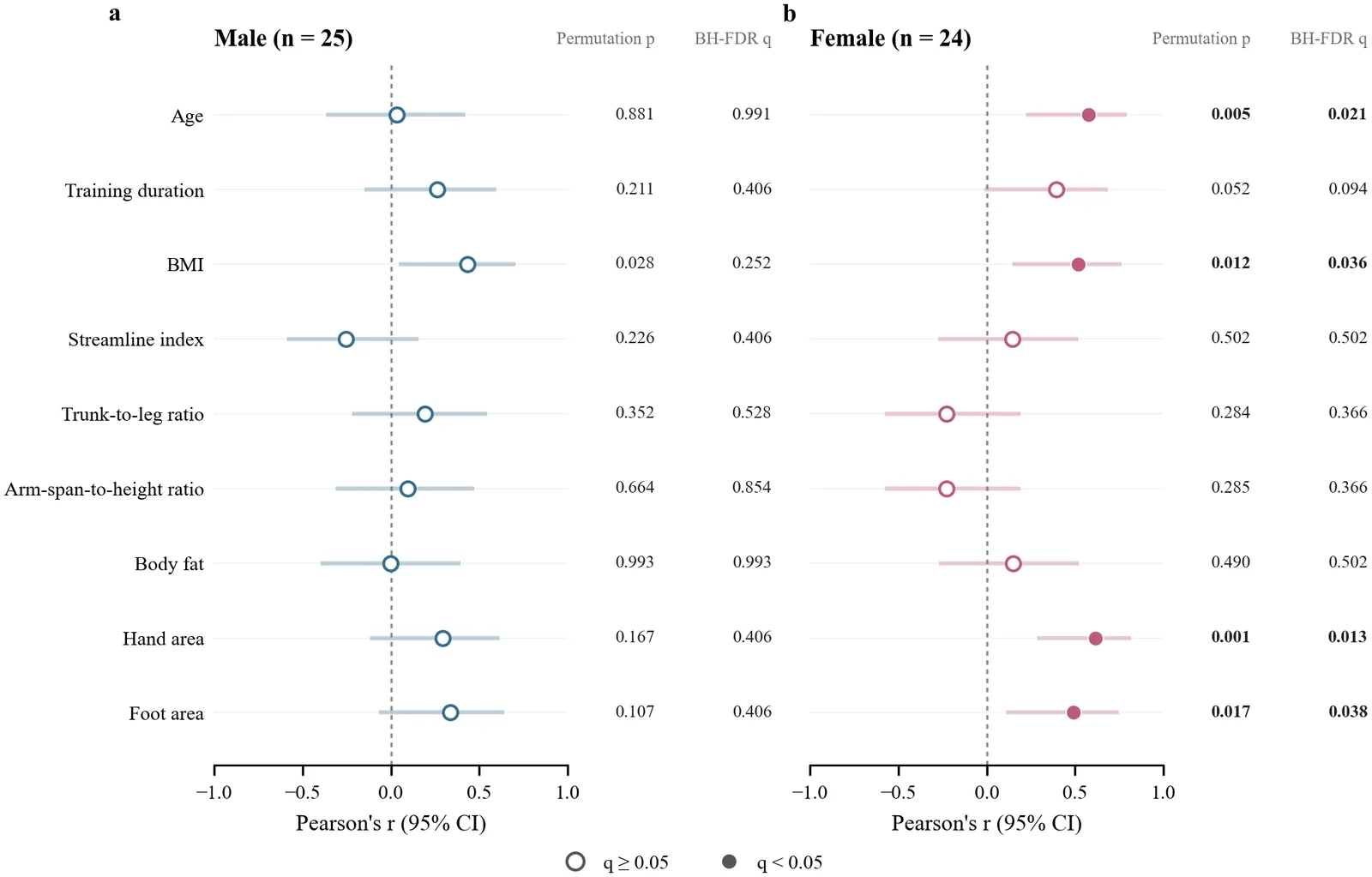
Pearson correlations of FINA points with body morphology and body composition measures in male and female swimmers. Points represent correlation coefficients, and horizontal lines represent 95% CIs based on Fisher’s z transformation. Permutation-test p values and within-group Benjamini-Hochberg FDR q values are listed on the right. Filled points indicate q < 0.05.

Among female swimmers, FINA points were positively correlated with age, BMI, hand area, and foot area after within-group FDR correction (**Figure 2b**). Spearman sensitivity analyses showed the same directions for these associations. Among male swimmers, BMI was positively correlated with FINA points at the uncorrected level, but the association did not remain significant after FDR correction. No other association survived FDR correction (**Figure 2a**). The corresponding sex-stratified scatterplots showed the same descriptive patterns and are provided in **Supplementary Figure 1**.

### 3.3 Sequential models and sex-stratified adjusted associations

**Figure 3:**
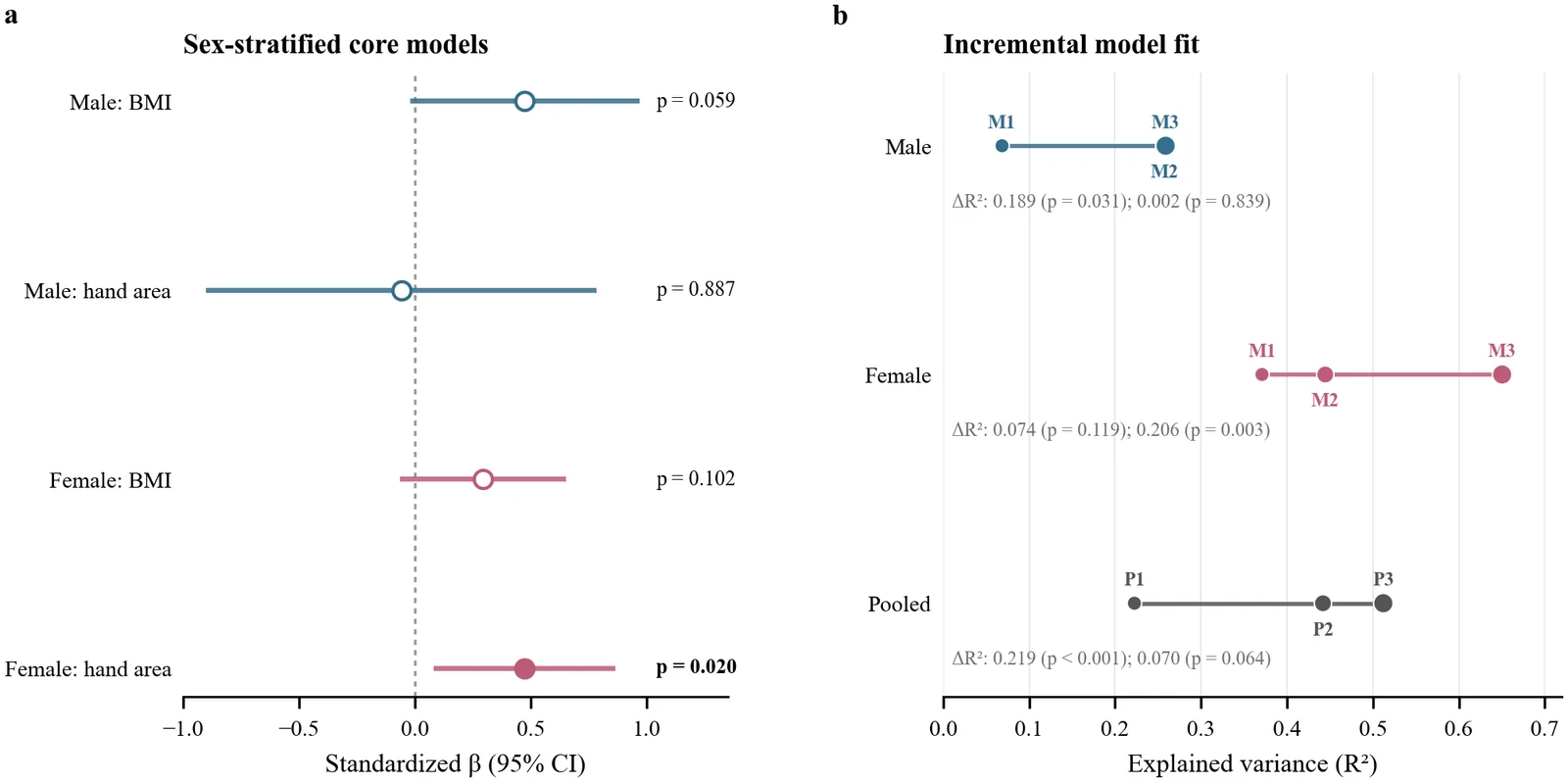
Core sex-stratified regression models and explained variance across sequential models. **(a)** Points represent standardised regression coefficients, and horizontal lines represent 95% CIs based on HC3 robust standard errors. **(b)** Points represent R² for each sequential model; the accompanying text reports ΔR² and the nested F-test p-value at each step.

Among male swimmers, adding BMI increased the explained variance at the corresponding model step, whereas adding hand area produced almost no additional explanatory value. Neither BMI nor hand area reached statistical significance in the final M3 model (**Tables 3**, **4**; **Figure 3**). Among female swimmers, adding hand area increased the explained variance by 20.6% (p = 0.003). In the final M3 model, hand area was positively associated with FINA points (β = 0.472, 95% CI [0.081, 0.863], HC3 p = 0.020), whereas BMI was not significant (**Tables 3**, **4**; **Figure 3**).

**Table 3:** Sequential linear regression models in sex-stratified and pooled samples.

Group	Model	n	R²	Adj. R²	ΔR² (p)	Model p	LOOCV RMSE
Male	M1 Age + training duration	25	0.069	-0.016	NA	0.458	69.2
Male	M2 + BMI	25	0.257	0.151	0.189 (0.031)	0.094	64.8
Male	M3 + hand area	25	0.259	0.111	0.002 (0.839)	0.179	69.5
Female	M1 Age + training duration	24	0.371	0.311	NA	0.008	80.1
Female	M2 + BMI	24	0.445	0.361	0.074 (0.119)	0.007	78.0
Female	M3 + hand area	24	0.650	0.577	0.206 (0.003)	< 0.001	62.5
Pooled	P1 Sex + age + training duration	49	0.223	0.171	NA	0.010	73.4
Pooled	P2 + BMI + hand area	49	0.442	0.377	0.219 (< 0.001)	< 0.001	65.5
Pooled	P3 + sex interactions	49	0.512	0.429	0.070 (0.064)	< 0.001	63.7

p-values for ΔR² were obtained from nested-model F-tests. LOOCV RMSE denotes the root mean square error from leave-one-out cross-validation. Sex interactions in pooled model P3 included only sex × BMI and sex × hand area.

**Table 4:** Coefficients from the core sex-stratified models and sex-interaction model.

Model	Variable	B	HC3 SE	Std. β	95% CI	p
Male M3	BMI	21.406	10.702	0.475	[-0.020, 0.970]	0.059
Male M3	Hand area	-0.412	2.871	-0.058	[-0.901, 0.785]	0.887
Female M3	BMI	18.972	11.052	0.294	[-0.064, 0.652]	0.102
Female M3	Hand area	2.839	1.123	0.472	[0.081, 0.863]	0.020
Pooled P3	Sex × BMI	-1.468	19.887	-0.019	[-0.540, 0.502]	0.942
Pooled P3	Sex × hand area	-54.699	37.472	-0.709	[-1.690, 0.272]	0.152

Coefficient p values and 95% CIs were based on HC3 heteroskedasticity-robust standard errors. Both the male and female M3 models were adjusted for age and training duration and included BMI and hand area. Pooled model P3 was additionally adjusted for the main effect of sex.

### 3.4 Pooled models, sex interactions, and robustness analyses

In the pooled sample, adding BMI and hand area to sex, age, and training duration increased the explained variance from 0.223 to 0.442 (ΔR² = 0.219, p < 0.001), while the LOOCV RMSE decreased from 73.4 to 65.5. Adding the sex × BMI and sex × hand area interactions resulted in a further increase of 0.070 in explained variance; however, the overall incremental contribution of the interaction block did not reach the conventional significance threshold (p = 0.064). Neither individual interaction was significant (sex × BMI: β = -0.019, HC3 p = 0.942; sex × hand area: β = -0.709, p = 0.152). The extended interaction screening of all nine measures likewise identified no sex-related moderating effects after FDR correction (all q > 0.05). The sex-stratified patterns should therefore be interpreted as descriptive findings and not as confirmed sex differences (**Tables 3**, **4**; **Figure 3b**).

The maximum VIF in the core models was 2.13, indicating no substantial multicollinearity. The Cook’s distance threshold of 4/n flagged 2 male and 3 female swimmers. After these observations were excluded, the BMI coefficient increased among male swimmers (β = 0.713, p < 0.001). Among female swimmers, the BMI coefficient was 0.386 (p = 0.012), and the hand-area coefficient decreased to 0.370 (p = 0.052). The direction of the hand-area coefficient remained positive, but its magnitude and statistical precision were sensitive to a small number of observations. These results indicate limited robustness and were therefore interpreted as a sensitivity analysis rather than confirmatory evidence.

## 4 Discussion

This exploratory study examined the associations of body morphology and body composition with each swimmer’s highest FINA point score in 49 provincial youth swimmers. FDR-corrected correlations were observed for age, BMI, hand area, and foot area among females, whereas no association survived correction among males. Hand area remained positively associated with FINA points in the primary adjusted female model, but the estimate was attenuated after influential observations were excluded. Formal interaction tests did not support sex-related moderation. Overall, the findings partly supported Hypotheses 1 and 2, whereas Hypothesis 3 was not statistically supported; all sex-stratified findings should be regarded as exploratory and hypothesis-generating.

The findings partly support Hypothesis 1, as associations between body morphology and body composition and competitive performance were observed mainly among female swimmers. In females, FINA points were positively correlated with age, BMI, hand area, and foot area after FDR correction. In males, only BMI reached uncorrected significance, and the association did not remain significant after correction. These findings suggest that physical structure may be related to standardised race performance within the age and performance ranges represented in this study. However, the associations were not consistent across all measures or both sexes, and they do not imply that increasing any single body dimension would necessarily improve competitive performance.

These findings are broadly consistent with the existing literature on youth swimming. A systematic review reported that height, arm span, body mass, and lean body mass were relatively consistently associated with stroke length, stroke index, and race performance in freestyle, whereas the evidence was substantially less consistent across other strokes (Alves et al., 2022). Lätt et al. found that arm span was an important anthropometric correlate of 100 m freestyle performance in male adolescent swimmers, although technical variables such as stroke index and stroke rate had greater explanatory value (Lätt et al., 2010). Moura et al. also observed associations of height, arm span, lean body mass, and upper-arm muscle area with upper-limb propulsive force in youth swimmers (Moura et al., 2014). More recent longitudinal research showed that hand area, arm span, in-water force, and swimming speed may change concurrently over a competitive season in young swimmers (Santos et al., 2024), while a study of prepubertal female swimmers reported an association between foot length and 50 m freestyle performance (Kuberski et al., 2025). Collectively, these studies and the present findings point to the relevance of body dimensions, while differences between studies suggest that age, event distance, stroke composition, and performance outcomes may shape the specific patterns of association.

The association between hand area and FINA points in female swimmers may reflect the mechanical relevance of effective propulsive surface area. Swimming propulsion is not determined by limb dimensions alone, but arises from the combined effects of hand surface area, relative water velocity, angle of attack, sculling direction, and technical timing. Computational fluid dynamics research has shown that hand trajectory and orientation are closely related to the pressure distribution, resultant force, and effective thrust acting on the hand (Sato and Hino, 2013). In trained swimmers, the use of larger hand paddles can reduce the energetic cost of swimming at a fixed speed, lower stroke rate, and increase stroke length, although the benefits diminish as paddle area increases (Crocker et al., 2021). A larger natural hand area may therefore provide a greater potential force-application surface during the catch and propulsive phases, but appropriate hand orientation, forearm coordination, and feel for the water remain necessary for this structural feature to translate into effective propulsion. The present association supports this structural basis but cannot substitute for direct hydrodynamic measurement. Foot area was also positively correlated with FINA points among female swimmers. Flutter kicking in freestyle and backstroke, dolphin kicking in butterfly, and the propulsive kick in breaststroke all require the feet and lower legs to alter water momentum; foot area may therefore be related to the size of the interface acting on the water. A review of flutter-kick propulsion indicated that lower-limb propulsion is associated with vortex formation and shedding, although direct evidence remains limited (Andersen and Sanders, 2018). Fin studies have shown that increasing the propulsive area at the distal foot can improve propulsive efficiency and alter distance travelled per kick (Zamparo et al., 2006). These findings provide indirect mechanistic support for a relationship between foot surface area and hydrodynamics, but fins and natural feet differ in shape, stiffness, and motor control. The foot-area finding should therefore be interpreted as a potential indication of swimming-specific structural advantage rather than as evidence of a direct effect of foot size on performance.

The positive association with BMI should be interpreted cautiously in the context of physical development in youth athletes. BMI is a composite measure of body mass relative to height and cannot distinguish muscle, bone, and adipose tissue. It also carries a substantial risk of misclassifying adiposity in adolescent athletic populations (Etchison et al., 2011). In the present study, BMI was associated with FINA points in female swimmers, whereas body fat percentage was not, suggesting that BMI may have reflected overall body size, lean-tissue accumulation, or maturational status more than adiposity itself. Similarly, research in youth swimmers has shown that height, arm span, body mass, and lean body mass may mediate relationships between biological maturation and propulsive force or sprint performance (Matos et al., 2025). BMI should therefore be regarded here as a broad indicator of physique and developmental status, and the present results do not support practical recommendations to increase body mass or alter body fat. Overall, Hypothesis 1 was supported for selected measures among female swimmers rather than universally. Swimming-specific anthropometric measures such as hand and foot areas may more closely reflect the structural demands of propulsion than general measures of body size.

Support for Hypothesis 2 was concentrated in the hand-area finding among female swimmers. After adjustment for age, training duration, and BMI, hand area remained independently and positively associated with FINA points (standardised β = 0.472, 95% CI [0.081, 0.863], HC3 p = 0.020). Adding hand area increased the explained variance by 20.6% (ΔR² = 0.206, p = 0.003), and the final model explained 65.0% of the variance in FINA points. By contrast, the adjusted association for BMI was not significant in the final model. Hand area therefore provided information beyond chronological age, training experience, and relative body mass in the female sample, consistent with the expectation in Hypothesis 2 that selected physical characteristics would have independent explanatory value. Age and training duration are important contextual variables in youth performance, but they capture different information. Chronological age is accompanied by changes in height, body composition, and neuromuscular development, whereas training duration may reflect the accumulation of technical practice, exposure to swimming-specific loads, and competition experience. Longitudinal research has shown non-linear relationships of maturational status with swimming velocity and stroke index in youth swimmers, while arm-propulsion efficiency remains independently associated with swimming speed beyond maturation (Abbott et al., 2021). Multiyear follow-up of talented youth swimmers has also shown that developmental trajectories in race performance, maximal swimming velocity, and stroke index are associated with the subsequent maintenance of a high competitive level (Post et al., 2024). Adjustment for age and training duration may therefore reduce evident confounding but cannot replace direct measures of biological maturation and technical quality.

The greater incremental explanatory value of hand area than BMI may reflect how closely each measure relates to the swimming-specific task. BMI is a general measure of physique composed of several tissue compartments, whereas hand area directly describes the potential surface through which the distal upper limb interacts with the water and is therefore more closely aligned with stroke propulsion. Research in youth swimmers has shown that height and body composition remain associated with upper-limb propulsive force after controlling for maturational stage (Moura et al., 2014), while seasonal tracking has placed changes in hand area within the same developmental process as in-water force, stroke kinematics, and performance (Santos et al., 2024). Considered alongside evidence from hand-paddle and fluid-dynamics studies (Crocker et al., 2021; Sato and Hino, 2013), the present findings suggest that morphological measures closer to the propulsive interface may characterise swimming-specific structural differences more sensitively than general relative body mass.

The adjusted association of hand area should be regarded as exploratory. Its inclusion was partly data-informed, the model was not preregistered or independently validated, and the limited subgroup size increased the risk of overfitting and optimistic estimates. After observations flagged by Cook’s distance were excluded, the coefficient remained positive but the p value increased to 0.052, indicating limited robustness. The present findings therefore suggest that swimming-specific anthropometric measures may offer explanatory value beyond conventional body-composition measures, but they do not justify a talent-identification threshold or an individual prediction tool.

The limited sex-specific sample sizes reduced statistical power, particularly for interaction testing, and increased the likelihood of false-negative findings, wide confidence intervals, and unstable estimates. FDR correction addressed multiple testing but could not eliminate instability arising from the small subgroups. The sex-stratified analyses showed different descriptive patterns of association, but the findings did not support Hypothesis 3. Age, BMI, hand area, and foot area were significantly correlated with FINA points among female swimmers, and hand area remained independently associated in the adjusted model. No association remained significant after FDR correction among male swimmers. In the pooled sample, however, adding the sex × BMI and sex × hand area interactions increased the explained variance by 7.0%, but the interaction block was not significant (p = 0.064). Neither the sex × BMI (p = 0.942) nor the sex × hand area (p = 0.152) interaction was significant, and none of the extended interaction tests across the nine measures survived FDR correction. A significant result in females and a non-significant result in males should therefore not be interpreted as evidence that the effects differ significantly between sexes.

Statistical power and the ranges of the analysed variables may help explain the different sex-stratified patterns. After stratification, the sample comprised only 25 males and 24 females, and small samples have particularly limited sensitivity for detecting interaction effects, as reflected by the wide CIs. In addition, provincial youth swimmers have undergone prolonged training and selection, which may restrict variation in body structure and competitive level. Hand area was less variable among male than female swimmers in the present sample, and this restriction of range may have attenuated correlations within the male group. The absence of significant associations among males may therefore reflect weaker underlying relationships, limited sample size, measurement error, or sample homogeneity; the current data cannot distinguish among these explanations.

Sex differences in the timing of pubertal maturation provide a biological context for potentially different association patterns, but chronological age cannot adequately characterise this process. Large competition datasets show that the performance gap between male and female youth swimmers begins to widen at approximately 10 years of age and overlaps with the pubertal period (Senefeld et al., 2019). Among female youth swimmers, menarcheal status is associated with the developmental trajectories of hand length, body size, strength, and performance across different distances (López-Plaza et al., 2025). Maturity-related selection bias may also be particularly evident at specific ages in female youth swimmers, and correction for maturational status can alter athlete-evaluation outcomes (Hogan et al., 2022). Because Tanner stage, years from peak height velocity, and menarcheal status were not measured in this study, the stratified differences may jointly reflect sex, maturational stage, and age structure.

Previous studies have not reached entirely consistent conclusions regarding sex-specific predictive patterns. Some have reported different combinations of anthropometric, physiological, and technical performance predictors according to sex and race distance in youth swimmers (Mezzaroba et al., 2013), whereas others have shown that most anthropometric and hydrodynamic differences between young male and female swimmers are markedly reduced after controlling for competitive performance (Barbosa et al., 2015). These discrepancies may reflect differences in performance level, stroke and distance, maturational stage, outcome definition, and model selection. The use of each swimmer’s highest FINA point score in the present study improved comparability across events but may also have obscured stroke- or distance-specific physical demands. Sex may therefore contribute to the relationship between physical characteristics and competitive performance, but the current evidence supports descriptive differences only. Confirmation requires larger samples and interaction tests that incorporate direct measures of maturation.

### 4.1 Limitations

First, the cross-sectional design meant that body morphology, body composition, and FINA points were obtained within the same observation period. The findings therefore identify associations but cannot establish temporal sequence or causal direction. Future studies should use longitudinal assessments at multiple time points to examine concurrent changes in growth, training adaptation, and race performance.

Second, the total sample was small, limiting the ability to detect small effects and interaction effects. The Cook’s distance sensitivity analysis also showed that several core coefficients were sensitive to a small number of observations. The observed effect sizes and model explanatory values therefore require replication in larger, multicentre samples of high-level youth swimmers.

Third, measures of maturation, including Tanner stage, years from peak height velocity, bone age, and menarcheal status, were unavailable. Chronological age and training duration capture only part of pubertal development, and residual confounding by maturation may have affected estimates of the associations of BMI and hand and foot areas with competitive performance. Adjustment for chronological age and training duration was therefore insufficient to fully account for inter-individual differences in biological maturation.

Fourth, selecting the highest FINA point score among each swimmer’s recorded events improved standardised comparability across events and distances, but a single maximum value cannot fully represent multi-event ability or preserve differences in propulsion and energetic demands among freestyle, breaststroke, backstroke, and butterfly. Future studies could stratify analyses by stroke, distance, and primary event and compare outcomes based on maximum points, mean points, and event-specific performance. Event-specific analyses were not performed because subdivision by sex, stroke, and distance would have yielded subgroups too small for reliable inference.

Fifth, body composition was represented only by body fat percentage. Other potentially relevant components, including lean mass and skeletal muscle mass, were not assessed; therefore, the study does not provide a comprehensive evaluation of body composition.

## 5 Conclusions

In this exploratory sample of provincial youth swimmers, age, BMI, hand area, and foot area were positively correlated with FINA points among females, whereas no association remained significant after FDR correction among males. Hand area showed a positive adjusted association in the primary female model, but the estimate was attenuated after influential observations were excluded. Formal interaction tests did not confirm sex-related moderation. These findings are hypothesis-generating and do not establish hand area as an independent predictor or talent-identification criterion. Larger longitudinal studies incorporating direct measures of maturation, event-specific performance, and hydrodynamics are required.

## Data Availability

The datasets presented in this study can be found in online repositories. The names of the repository/repositories and accession number(s) can be found in the article/**Supplementary Material**.
