# Supplementary material for: Associations of body morphology and body composition with swimming performance in male and female youth swimmers: an exploratory cross-sectional study
Source: Front Physiol. 2026 Sep 10;17:1963914. doi: 10.3389/fphys.2026.1963914 (PMC13600859; doi:10.3389/fphys.2026.1963914)
Supplement: Supplementary file 1 [file SupplementaryFile1.docx]

**Supplementary Material**

**Supplementary Figure S1.** Sex-stratified scatterplots of BMI and hand area against FINA points. Solid lines represent ordinary least-squares linear fits, and shaded bands represent 95% CIs for the mean response. p-values were obtained from two-sided permutation tests, and q-values are within-group Benjamini-Hochberg FDR-adjusted values.
